# Signal Detection Using Change Point Analysis to Identify Safety Signals from Spontaneous Reports in Aortic Stent Grafts in Japan

**DOI:** 10.1007/s10916-026-02388-z

**Published:** 2026-04-23

**Authors:** Kohei Takahashi, Hideto Yokoi, Fumiaki Mikami, Tomomi Satomi, Morikazu Seki, Takako Takayama Niwa, Hideki Hanaoka, Yosuke Inaba, Daisuke Koide

**Affiliations:** 1 Biostatistics section, Clinical Research Center, Chiba University Hospital, Chiba, Japan; 2 Department of Medical Informatics, Kagawa University Hospital, Kagawa, Japan; 3 Clinical Research Support Center, Kagawa University Hospital, Kagawa, Japan; 4 Division of Medical Safety and Report Management Office of Vigilance and Standards for Medical Devices, Pharmaceuticals and Medical Devices Agency, Tokyo, Japan; 5 Clinical Research Center, Chiba University Hospital, Chiba, Japan; 6 Department of Biostatistics and Bioinformatics, Graduate School of Medicine, The University of Tokyo, Tokyo, Japan

**Keywords:** Signal detection, Change point analysis, Spontaneous report, Medical device, Device malfunction

## Abstract

Signal detection is essential for identifying potential safety issues with drugs and medical devices. Change point analysis (CPA) detects changes in longitudinal data for a medical device without incorporating comparisons with other devices. This study investigated the temporal relationship between endoleak reports for aortic stent grafts identified using CPA and the issuance of the “Pharmaceuticals and Medical Devices Agency (PMDA) Alert for Proper Use of Medical Devices” in Japan. Device malfunction and adverse event reports for aortic stent grafts submitted to the PMDA between April 2008 and September 2022 were analyzed. The reports were mapped to the Medical Device Problem Terminology from the Japan Federation of Medical Devices Associations. CPA was used to identify temporal changes in report counts, with change points having confidence levels ≥ 90% being considered significant. A total of 20,678 reports were analyzed, including 23,541 device malfunctions and 30,500 adverse events. Endoleaks accounted for 10,779 events. CPA identified a significant increase in endoleak events in April 2015 (2 years before the regulatory alert). Confidence levels exceeded 90% in 76 out of 174 evaluated time points, first in July 2008 (9 years before the alert) and next in April 2016 (1 year before the alert). Based on these findings, CPA may help prioritize investigations of medical-device-related malfunctions and adverse events. However, the results do not indicate earlier or superior signal detection relative to regulatory authorities. Further research is needed to assess CPA’s applicability to other medical devices and malfunctions.

## Introduction

In the context of pharmacovigilance, signal detection is a method for prioritizing further investigation of potential safety signals (defined as information suggesting a possible causal relationship between a drug and an adverse event that is previously unknown or not yet fully substantiated) [1, 2]. To perform drug signal detection using spontaneous reporting databases, a two-by-two contingency table is often created by selecting a specific drug–event combination and comparing the number of events for that combination versus all others [3]. The reporting odds ratio (ROR) [4], proportional reporting ratio (PRR) [5], and Bayesian confidence propagation neural network (BCPNN) method [6] are some of the commonly used signal detection indices. Although there is no universal gold standard for signal detection indices because of differences in database structures across countries [7] and the inherent nature of adverse events, ROR and PRR are commonly used to monitor safety signals [8].

Change point analysis (CPA) is a statistical method used to detect points in time where longitudinal data trends significantly change [9–11]. CPA and signal detection are both used as data mining techniques to identify relevant associations or combinations that warrant further investigation [12]. Compared with conventional signal detection methods, CPA does not require contingency tables and can analyze changes in the number of reported adverse events or malfunctions for a single device. Although it was originally developed for use in engineering and economics [13], CPA has increasingly been applied in the medical field [14, 15]. In public health, CPA has also been used as a complementary tool for signal detection with post-marketing surveillance data from databases, including the US Food and Drug Administration’s Manufacturer and User Facility Device Experience database. CPA has also been applied to examine events associated with neurostimulator devices that led to treatment discontinuation [16] and to evaluate syncope events following human papillomavirus vaccination using the Korea Adverse Event Reporting System (KAERS) database [15]. In a previous study, CPA was applied to events of benfluorex-associated aortic valve incompetence. Data were obtained from the French Base Nationale de Pharmacovigilance and European EudraVigilance databases. These applications demonstrated the utility of CPA in detecting changes in adverse event reporting over time [17]. Based on these findings, CPA can serve as a complementary tool to traditional signal detection methods in monitoring medical devices and drugs.

For medical devices, signal detection indices similar to those used for drugs are applied. However, several challenges limit their applicability. First, the slower development of monitoring systems and corresponding databases has hindered the establishment of robust safety surveillance infrastructure [18, 19]. Furthermore, the absence of international guidelines for reporting terminology has delayed the harmonization and adoption of consistent signal detection practices [20, 21]. Second, frequent modifications and improvements in device design make continuous evaluation more challenging. Device-specific issues, including malfunctions influenced by operator or user factors, can further complicate assessment [18, 20]. Third, rare or unique malfunctions not observed in other devices can result in “zero” cells in contingency tables, making the calculation of indices such as ROR difficult [20, 22]. Thus, the conventional signal detection approaches developed for drugs may not be directly applicable to medical devices, and standardized methodologies for medical device surveillance remain underdeveloped. Considering that spontaneous data are now gradually accumulating for medical devices [23], approaches that capture temporal changes in reporting—rather than solely relying on single-timepoint analyses—are increasingly important for effective signal detection. In this context, CPA is intended to support the prioritization of device–malfunction combinations, helping to identify those that warrant further clinical or regulatory review.

Several countries have established reporting systems for collecting safety information on drugs, medical devices, and regenerative medicine products [24]. In Japan, manufacturers, healthcare professionals, and other relevant parties are required to report to the Pharmaceuticals and Medical Devices Agency (PMDA) under the Act on Securing Quality, Efficacy and Safety of Products Including Pharmaceuticals and Medical Devices [25]. Although the PMDA signal management process is not fully transparent, it is understood to incorporate multiple data sources, including spontaneous reports, published literature, and information from other regulatory authorities. While quantitative signal detection methods may contribute to internal evaluations, the specific methodologies and their relative roles are not publicly disclosed.

The PMDA issues a “PMDA Alert for Proper Use of Medical Devices” to provide further guidance to healthcare professionals when certain device malfunctions, infection events, or other safety-related events continue to be reported despite prior safety alerts. Although this alert system is specific to Japan, other countries also have mechanisms for disseminating medical device safety information [26]. In July 2017, an alert was issued in Japan for aortic stent grafts used in endovascular aortic repair [27] following a series of complication events, including endoleaks, stenosis, and infected aneurysms [28]. These complications can stem from various causes (e.g., off-label use and inadequate procedural skill) [29, 30] and are not necessarily because of inherent product defects.

Therefore, the decision to issue such a request is based not only on quantitative criteria (e.g., an increase in reported events) but also on qualitative assessments by medical experts. Importantly, the timing of a publicly issued safety alert reflects not only potential signal identification but also subsequent expert review, regulatory decision-making, and administrative procedures required for communication. According to previous studies, regulatory processes involve multiple stages following signal detection, with variable time intervals between detection and subsequent actions [31]. Therefore, the alert date should not be interpreted as the true timing of signal detection, but rather as a regulatory milestone influenced by multiple procedural steps. Moreover, it should not be considered as a direct comparator for statistical signal detection methods.

The present study aimed to examine the temporal relationship between endoleak events associated with aortic stent grafts identified using CPA and the issuance of the “PMDA Alert for Proper Use of Medical Devices.” This regulatory alert was treated solely as a contextual milestone for comparing the timing of change points and should not be interpreted as evidence that CPA enabled earlier detection than the regulatory authority. Endoleak was selected for analysis as it represents a significant complication associated with aortic stent grafts [27, 32]. Considering the lack of a gold standard and the absence of universally accepted quantitative indices for signal detection in medical devices, this study investigated whether CPA could assist in prioritizing investigations of device malfunctions or adverse events associated with medical devices.

## Methods

### Data Source

This study analyzed device malfunctions and adverse event reports related to medical devices that were submitted to the PMDA by manufacturers between April 1, 2008, and September 30, 2022. For analysis purposes, the unit of submission to the PMDA was defined as a “report,” corresponding to one individual report, whereas each device malfunction or adverse event recorded within a report was treated as an individual “event,” considering that multiple events may occur per report. Individual report-level data were obtained from the PMDA through research collaboration. Specifically, this study focused on reports wherein the medical device’s generic name was listed as “aortic stent graft.” If the same report was submitted multiple times, only the first report was included in the analysis. Subsequent reports and any withdrawn reports were excluded.

### Mapping of Report Terminology

**Fig. 1:**
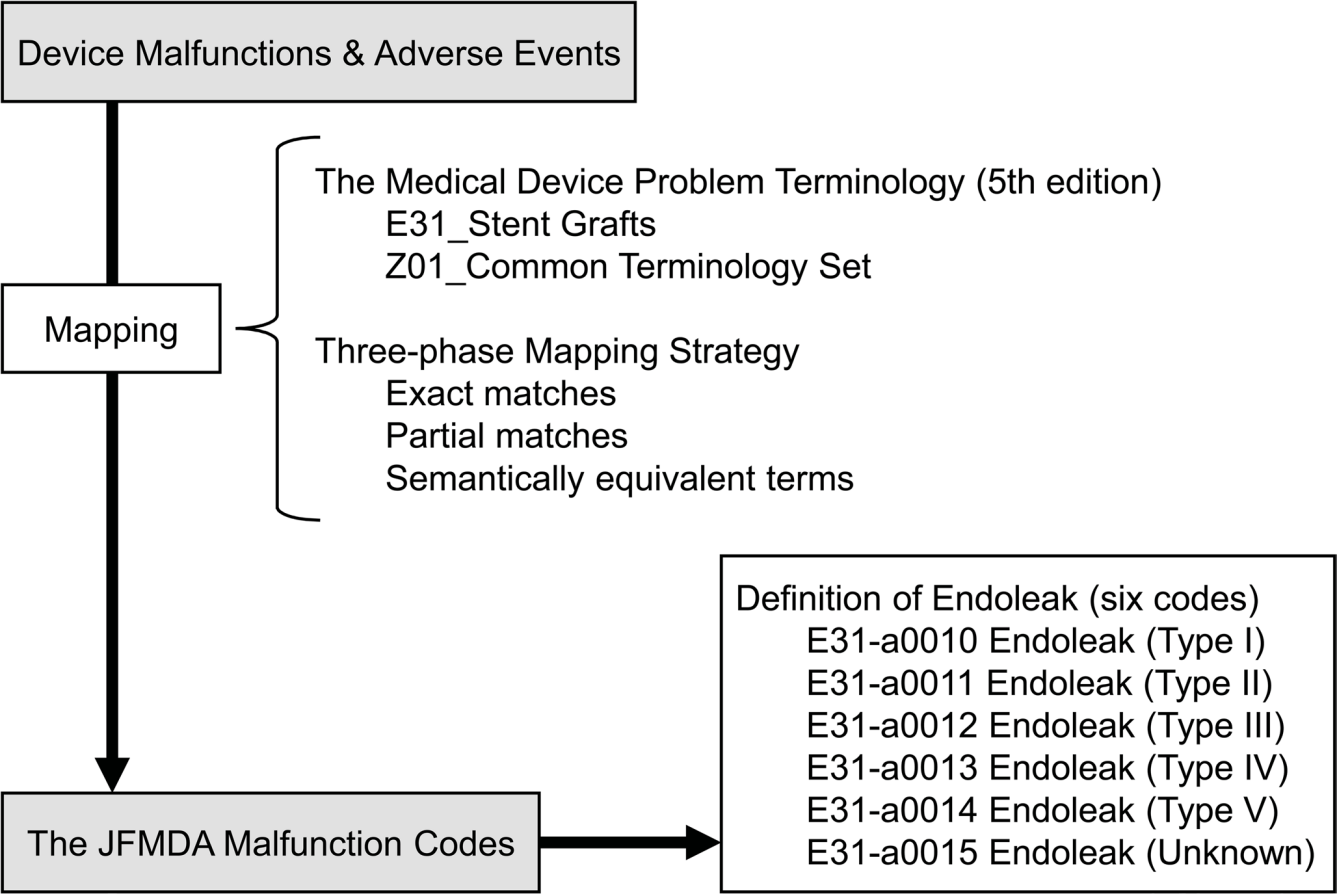
Workflow of the terminology mapping and classification process. Abbreviation: JFMDA, Japan Federation of Medical Devices Associations

The overall mapping process is shown in Fig. 1. Device malfunctions and adverse events from individual events were mapped to the Medical Device Problem Terminology (5th edition) from the Japan Federation of Medical Devices Associations (JFMDA) [33] using the terms listed under “E31_Stent Grafts” and “Z01_Common Terminology Set.”

Following a three-phase mapping strategy [34], the mapping process classified all events according to the device malfunction and adverse event terminology defined in the glossary by systematically extracting exact matches, partial matches, and semantically equivalent terms. Terms that did not correspond to any of these categories were defined as unclassifiable. Endoleak was defined as any event categorized under one of the following six codes based on the JFMDA malfunction codes: E31-a0010 Endoleak (Type I), E31-a0011 Endoleak (Type II), E31-a0012 Endoleak (Type III), E31-a0013 Endoleak (Type IV), E31-a0014 Endoleak (Type V), and E31-a0015 Endoleak (Unknown).

### Statistical Analysis

CPA is a nonparametric method proposed by Taylor [35] that estimates the timing and statistical significance of change points in time-series data using cumulative sums (CUSUM) and bootstrap resampling [36]. For each time point $\:i$ (with a total of $\:n$ time points), the number of events is denoted as $\:{X}_{i}$. Then, CUSUM $\:{S}_{i}$ is calculated as follows (with $\:{S}_{0}=0$):

1 $$S_i=S_{i-1}+\left(X_i-\frac1n\sum\:_{i=1}^nX_i\right).$$

The difference between the maximum and minimum values of $\:{S}_{i}$ over all time points is defined as follows:

2 $$S_{diff}=\underset{i=0,\:\dots\:,\:n}{\mathrm{max}}S_i-\underset{i=0,\:\dots\:,\:n}{\mathrm{min}}S_i.$$

To evaluate the confidence level of a potential change point, the time series is randomly permuted $\:k$ times (with $\:k=\mathrm{1,000}$ in this study) to generate bootstrap samples. For each sample, $\:{S}_{diff}^{k}$ is calculated. The confidence level, which represents the statistical confidence in the observed trend, is defined as follows:

3 $$Confidence\:level\:\left[\%\right]=\frac Qk\times\:100.$$

where $\:Q$ is the number of bootstrap samples for which $\:{S}_{diff}^{k}<{S}_{diff}$. A confidence level ≥ 90% is considered significant [35]. Based on the CUSUM sequence $\:{S}_{i}$, the change point is estimated at time $\:m+1$, where $\:m$ is the index of the largest absolute CUSUM value:

4 $$S_m=\underset{i=0,\:\dots\:,\:n}{\mathrm{max}}\left|S_i\right|.$$

For CPA, events were aggregated by month based on the reporting date to the PMDA. Each month was treated as a single time point, which corresponds to 174 months between April 2008 and September 2022. Moreover, sensitivity analysis was performed using quarterly aggregation, which corresponds to 58 time points over the same study period. All statistical analyses were performed using SAS software (version 9.4, SAS Institute, Cary, NC, USA).

## Results

A total of 20,678 reports related to aortic stent grafts were submitted to the PMDA. Among them, 23,541 and 30,500 events were reported as device malfunctions (including multiple events per report) and adverse events (including multiple events per report), respectively.

A breakdown of endoleaks that were reported as device malfunctions is shown in Table 1. As shown in the table, there were 7,701 endoleak events, accounting for 32.7% of all device malfunction events. Moreover, 3,078 endoleak events were reported as adverse events rather than device malfunctions, resulting in a total of 10,779 endoleak events when these are included. Among device malfunction events, 7,068 events (30.0%) were reported as “unknown,” 600 events (2.5%) were classified as adverse events without any associated device malfunction, and 1,086 events (4.6%) were categorized as “unclassifiable” because they could not be assigned to any term in the glossary.

**Table 1:** Breakdown of device malfunctions for Endoleaks

Name of Device Malfunction	JFMDA Malfunction Codes	Number of Events	Proportion
Endoleak (Type I)	E31-a0010	5,399	70.1%
Endoleak (Type II)	E31-a0011	529	6.9%
Endoleak (Type III)	E31-a0012	1,037	13.5%
Endoleak (Type IV)	E31-a0013	110	1.4%
Endoleak (Type V)	E31-a0014	96	1.2%
Endoleak (Unknown)	E31-a0015	530	6.9%
Endoleak (Total)	-	7,701	-

**Fig. 2:**
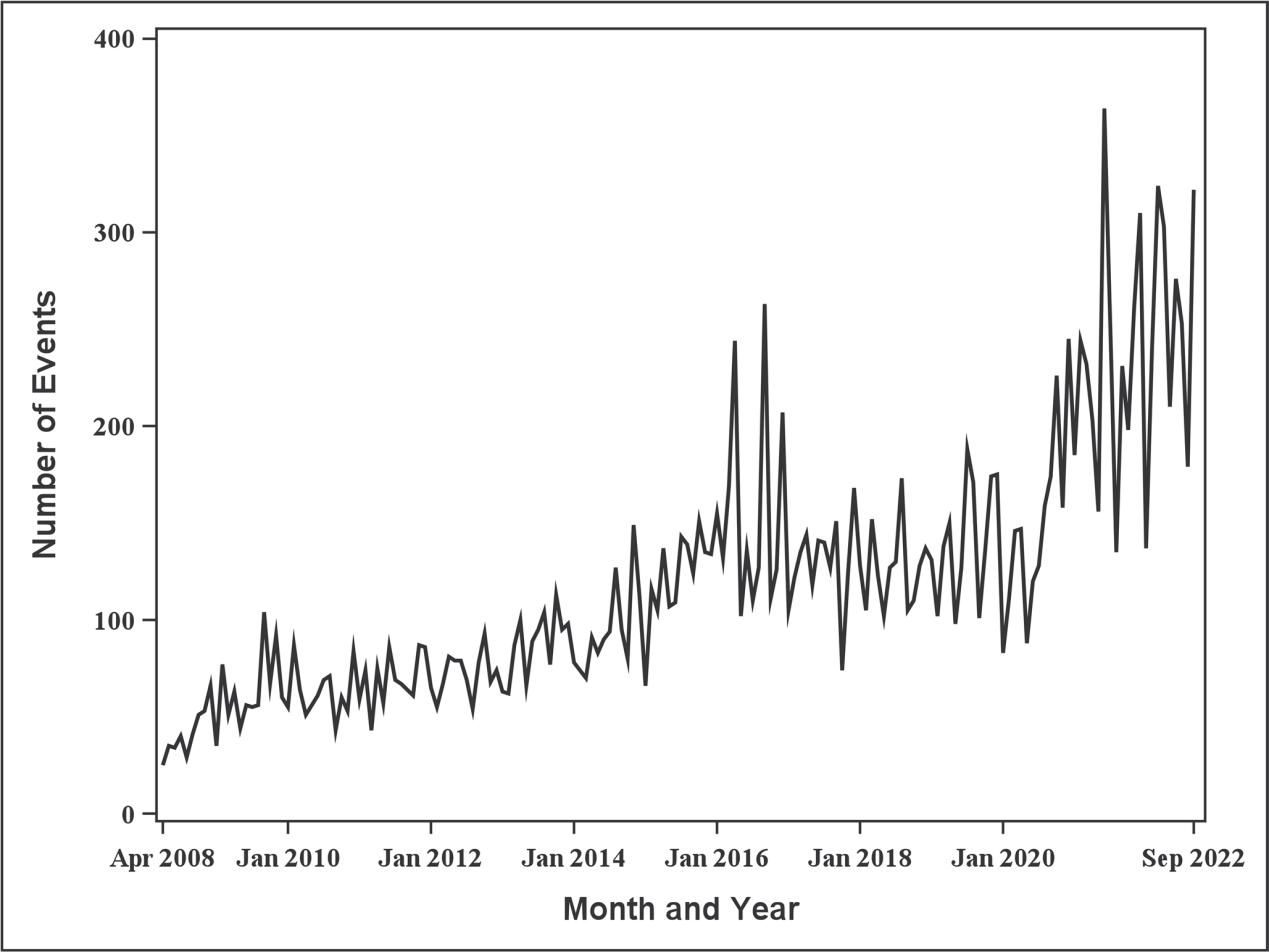
Trends in the number of device malfunctions events for aortic Stent grafts by month

As shown in Fig. 2, the monthly number of device malfunction events related to aortic stent grafts demonstrated an overall increasing trend over time. The monthly number of events increased from 25 in April 2008 (start of data aggregation) to 322 in September 2022, corresponding to a nearly 13-fold increase.

**Fig. 3:**
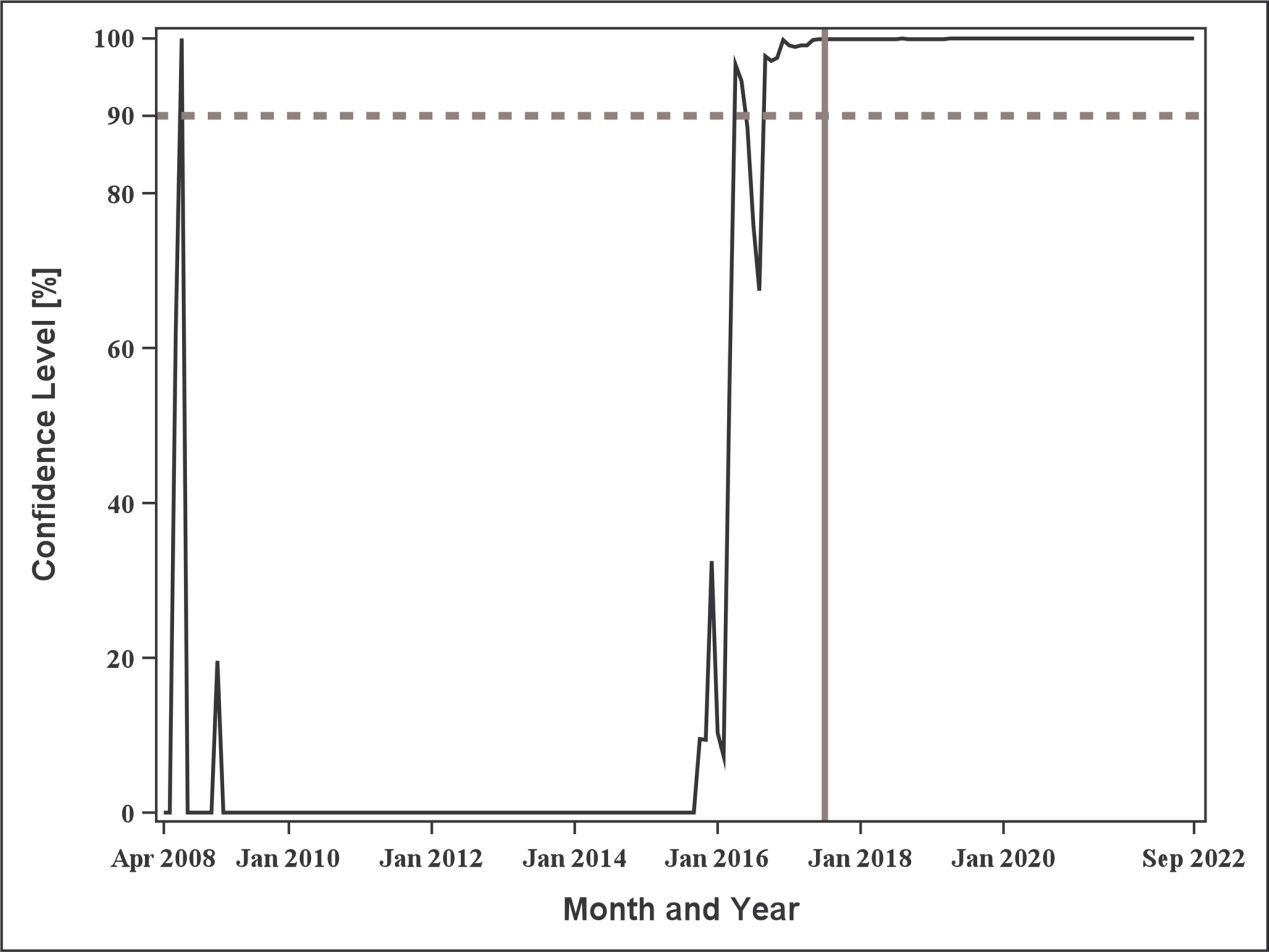
Trends in confidence levels for Endoleak events by month. The dotted line represents 90% of the threshold, whereas the solid line indicates the timing of the “PMDA Alert for proper use of medical devices” (July 2017)

The sequentially estimated confidence levels were obtained when CPA was applied to the monthly number of endoleak events (Fig. 3). The confidence level exceeded 90% in July 2008, April and May 2016, and September 2016 to September 2022, corresponding to a total of 76 time points above the 90% threshold. As shown in the figure, the confidence level exceeded the 90% threshold for the first time in July 2008. However, this point corresponded to only the fourth time point after the start of data aggregation (April 2008). This means that even minor fluctuations could result in a large increase in confidence levels because of the small number of observations. Furthermore, the confidence level dropped to 0% in the subsequent month. This finding suggests that a meaningful change was unlikely to have occurred at that time. From April 2016 onward, confidence levels consistently exceeded 90%. This indicates the possibility that a significant change occurred during this period.

**Fig. 4:**
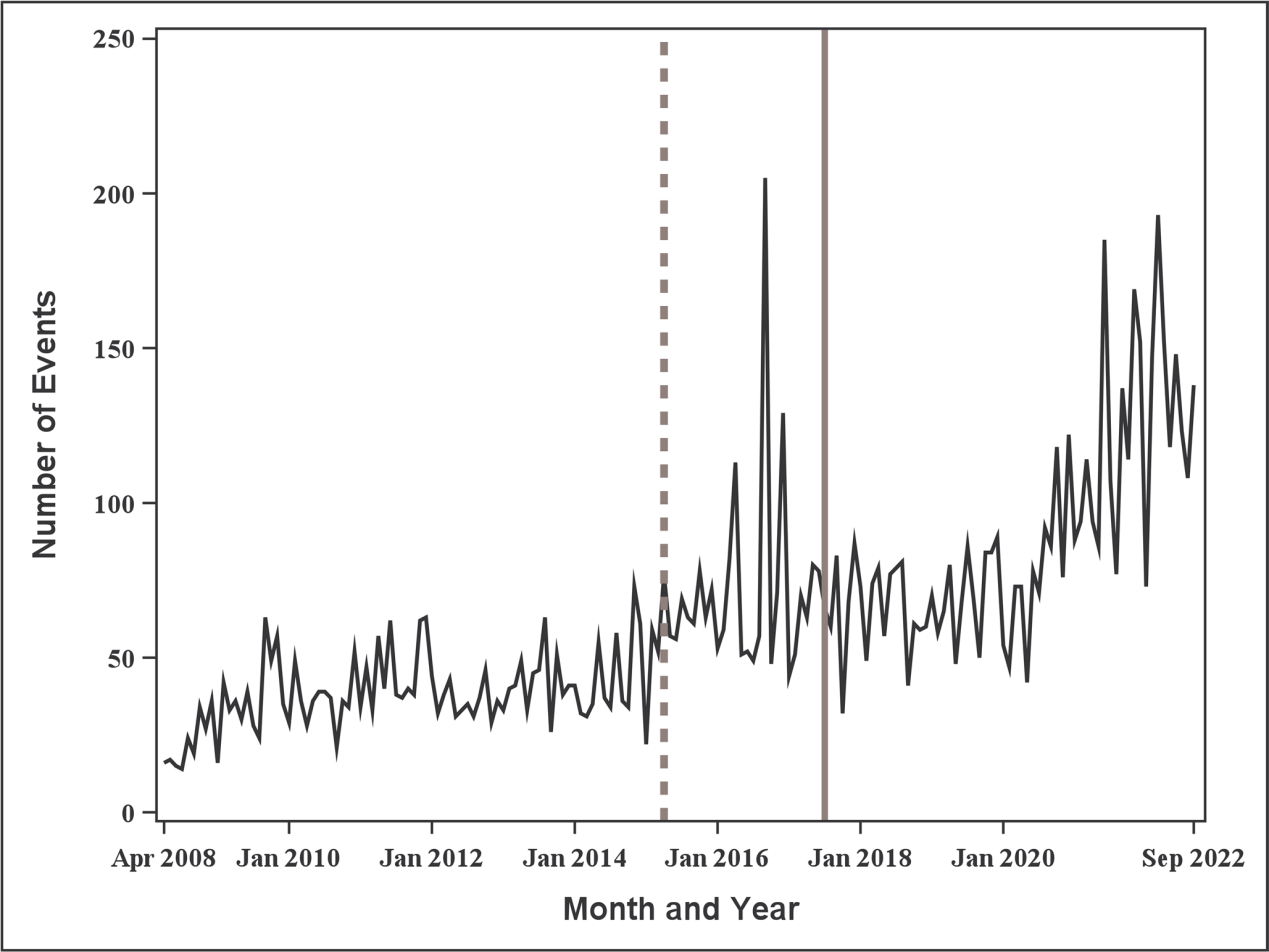
Trends in the number of Endoleak events for aortic Stent Grafts by month. The dotted line indicates the change point, whereas the solid line indicates the timing of the “PMDA alert for proper use of medical devices” (July 2017)

The monthly number of endoleak events is shown in Fig. 4. As can be seen, the monthly number of endoleak events increased from 16 at the beginning of the observation period to 138 at the end, corresponding to an approximately 8.6-fold increase. A change point was detected in April 2015, with the mean monthly number of events increasing from 38.6 (standard deviation: 12.4) before the change point to 83.7 (standard deviation: 35.1) after the change point.

The first month wherein the confidence level exceeded 90% (around April 2016) and the detected change point (April 2015) both occurred prior to the issuance of the “PMDA Alert for Proper Use of Medical Devices” in July 2017. Thus, the temporal increase in the monthly number of endoleak events preceded the regulatory alert by approximately one to 2 years.

**Fig. 5:**
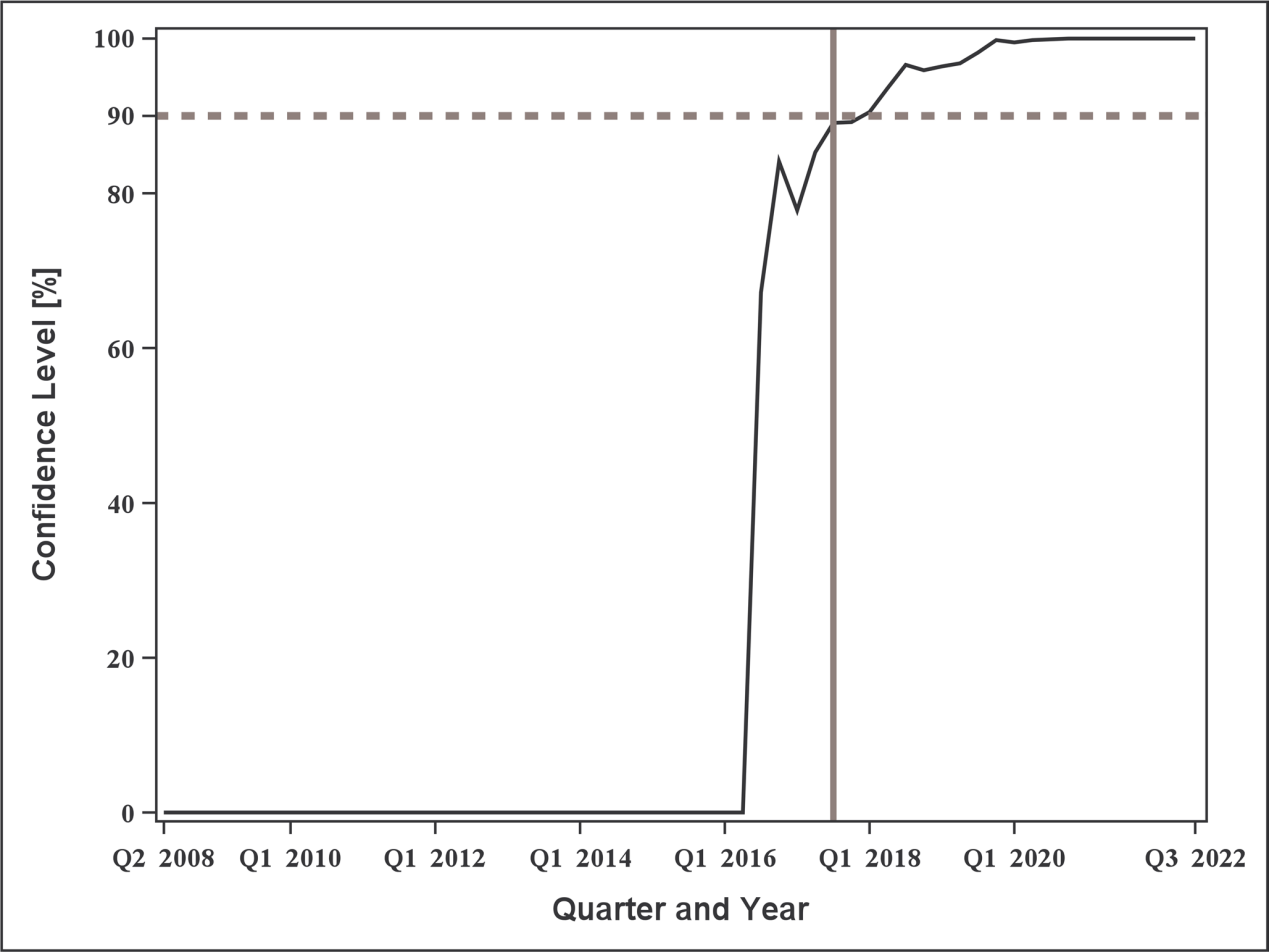
Trends in confidence levels for Endoleak events by quarter. The dotted line represents 90% of the threshold, whereas the solid line indicates the timing of the “PMDA alert for proper use of medical devices” (Q3 2017)

**Fig. 6:**
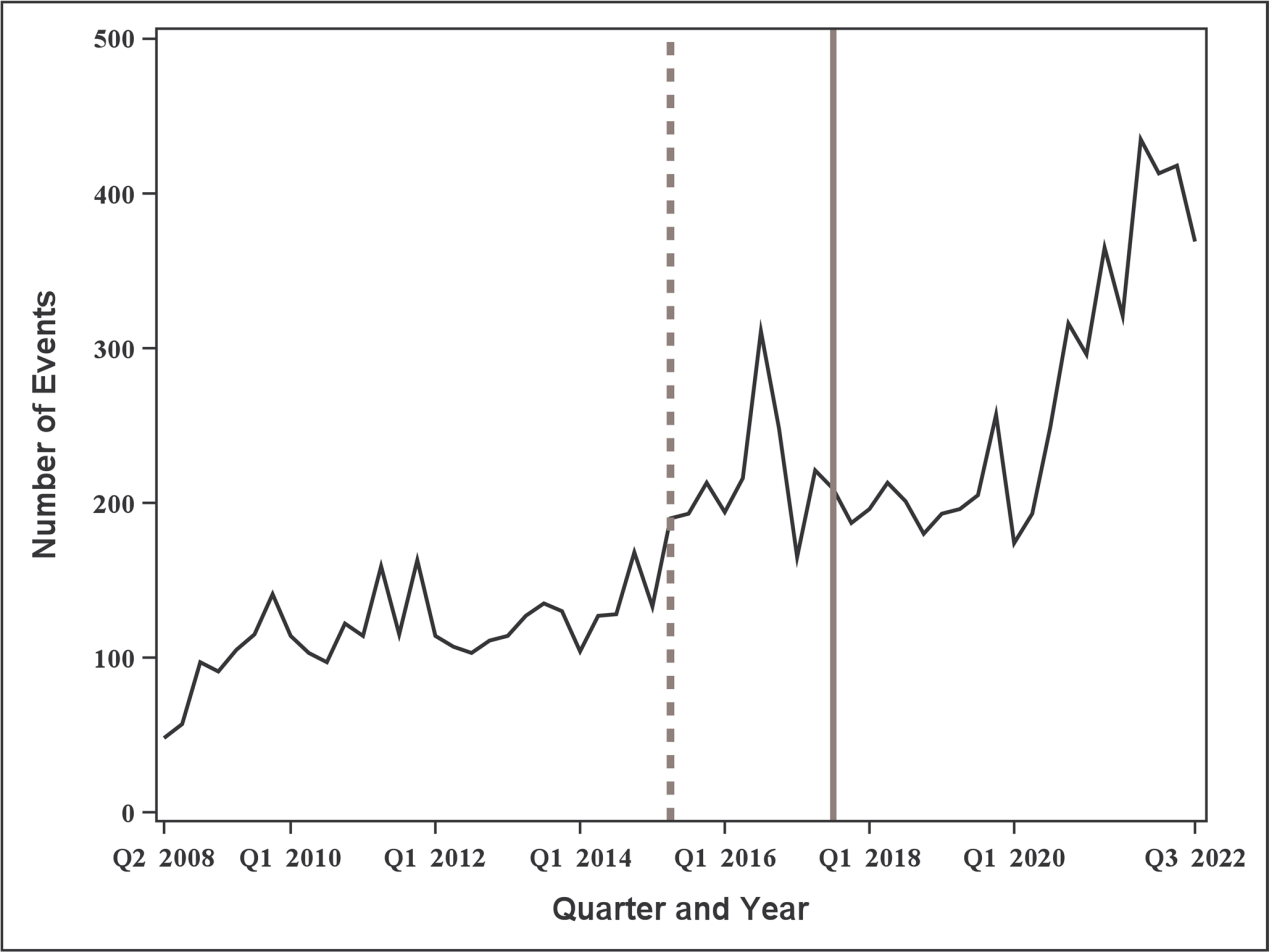
Trends in the number of Endoleak events for aortic Stent grafts by quarter. The dotted line indicates the change point, whereas the solid line indicates the timing of the “PMDA alert for proper use of medical devices” (Q3 2017)

In the sensitivity analysis, the confidence level exceeded 90% from Q1 2018 to Q3 2022, corresponding to a total of 19 time points above the 90% threshold (Fig. 5). As shown in Fig. 6, a change point was detected in Q2 2015, with the mean quarterly number of events increasing from 115.8 (standard deviation: 25.9) before the change point to 251.2 (standard deviation: 78.9) after the change point. These results were largely consistent with the findings of the main monthly analysis.

## Discussion

In this study, the sequential estimation of confidence levels and change point detected increases in endoleak events related to aortic stent grafts by approximately 1–2 years before the issuance of the regulatory alert. This indicates that CPA may serve as a complementary tool for monitoring temporal changes in adverse event reporting. However, these results should not be interpreted as evidence of earlier or superior signal detection relative to the regulatory authority as CPA does not directly capture the risk thresholds underlying regulatory alert issuance. These findings are consistent with those of previous studies that applied CPA to pharmacovigilance data, which found that CPA can identify change points in reporting trends that may correspond to emerging safety signals [15–17]. In another study that examined vaccine safety data from the KAERS database, CPA effectively detected increases in adverse events, although its performance may be limited when the number of events is small [15]. This limitation is further illustrated in the early-period observations (e.g., July 2008) in the present study, where confidence levels were less stable. Taken together, the findings of the present study and previous research indicate that CPA can provide valuable complementary information for detecting temporal increases in adverse events across different types of medical products, including pharmaceuticals and medical devices.

Regarding the observation that significant confidence levels were obtained for all time points from September 2016 onward, the significant change point in the first half of 2016 may have continued to influence confidence levels when data up to each subsequent point were included, thereby maintaining the increased confidence levels. Distinguishing whether the increased confidence levels are attributed to the influence of an earlier change point or the occurrence of a new significant change is difficult. To address this issue, limiting the calculation to a fixed number of preceding time points, instead of using all available data before each evaluation point to compute the confidence level, may help reduce the persistent influence of earlier significant change points.

As shown in Fig. 4, April 2015 was detected as the change point. This indicates that the number of device malfunctions had begun to increase approximately two years before the issuance of the regulatory alert in July 2017. Moreover, it suggests that a change in reporting occurred around this point. These findings were consistent with those of sensitivity analysis, wherein a similar change point was observed in Q2 2015. In this study, the change point for endoleak events corresponded to increases. However, decreases could also be detected in principle. In signal detection of device malfunctions or adverse events, priority is given to detecting change points associated with increases rather than decreases. Therefore, when estimating the change point using the CUSUM estimator, replacing Eq. (4) with Eq. (5) allows the estimator to be modified to detect only increasing change points:

5 $$S_m=\underset{i=0,\:\dots\:,\:n}{\mathrm{max}}S_i.$$

By calculating $\:m$ and $\:m+1$ using Eq. (5), it may be possible to efficiently detect only the time points at which increases occur. One important consideration in evaluating the timing of change points is that the CUSUM estimator depends on the range of data used. As indicated in Eq. (1), the estimator uses the overall mean of all time points. This means that the estimated change point is specific to the selected data range. Expanding or narrowing the range will shift the estimated change point. If CPA is to be applied for signal detection in practice, real-time monitoring of reporting frequency is essential to detect increases as they occur. Consequently, the retrospective evaluation of change points estimated by the CUSUM estimator may not align well with the purpose and role of signal detection.

This study has several limitations. In pharmacovigilance, signal detection indices are often used to determine whether a particular drug has a relatively high number of events compared with others [3–6]. However, CPA only evaluates the number of events for a specific medical device and specific adverse event or device malfunction without incorporating comparisons with other medical devices or the characteristics of spontaneous reporting data. Therefore, it should not be considered as an equivalent to signal detection indices, including ROR, PRR, or BCPNN. It is more appropriate to employ CPA as a complementary tool in combination with other methods within a broader signal detection framework [17], rather than determining whether CPA is superior to these conventional methods. Further investigations are needed to determine its practical utility.

Spontaneous reporting data are known to be biased toward serious, unknown, or highly publicized events, which tend to be reported more frequently. By contrast, common or familiar events are often underreported. Information disseminated by the PMDA, manufacturers, or mass media may lead to overreporting [37–39]. Regarding the number of endoleak events, the increase in the number of events after 2017 may have been influenced by the dissemination of the regulatory alert, which could have caused overreporting. However, directly attributing the increase to the alert is extremely difficult. Furthermore, the overall use of aortic stent grafts has been increasing (Fig. 2), and data on device usage or sales were not available in the present study. In comparing Fig. 2 with Fig. 4, the increased use of devices rather than an event-specific rise is likely the primary driver of the observed increase in endoleak events.

The evaluation of CPA may be challenging when applied to rare or late-onset device malfunctions or adverse events. In a previous study using CPA [15], the sensitivity of detecting change points increased as the average number of events increased. Therefore, further studies are needed to determine whether CPA can be appropriately applied to rare device malfunctions or adverse events with few reports, or to newly developed medical devices with a limited number of reporting time points. Moreover, CPA may be unstable in short time series and when the number of events at each time point is small. In such cases, random variation can have a greater impact on the CUSUM statistic, and the bootstrap procedure may not provide a reliable reference for expected fluctuations. Consequently, some detected confidence levels or change points may represent stochastic variation, rather than true structural changes. As shown in Fig. 3, the confidence level exceeded 90% shortly after data aggregation began (July 2008), despite the very limited number of observations at that stage. Therefore, the results should be interpreted with caution. Conversely, this early-time-point artifact was not observed in the sensitivity analysis using quarterly aggregation, which may be because of the increased number of events per time point (Fig. 5). Based on these findings, the choice of temporal aggregation interval may influence the stability of CPA. Further investigations are needed to determine the appropriate time interval for its application.

In pharmacovigilance, signal detection may fail for late-onset adverse events [39], and a similar limitation may apply to medical devices if device malfunctions or adverse events do not immediately occur after use. In the case of implantable devices, immediate removal or replacement may pose a higher risk to the patient even if a device malfunction or adverse event occurs [40, 41]. Consequently, reports may be submitted when the device is eventually removed, leading to a discrepancy between the event occurrence date and the report or cause identification date. Therefore, the reporting date to the PMDA does not necessarily coincide with the actual occurrence date of device malfunction or adverse event. Moreover, the dataset used in this study may have included events that occurred prior to the reporting date.

Taken together, these considerations highlight key limitations that should be taken into account when applying CPA in practice, including reporting bias, temporal changes in device utilization, and difficulty of distinguishing genuine increases in incidence from variations in reporting behaviors.

There is also room for improvement in the detailed classification and mapping of the data used. Although collecting information about the cause is desirable, 30.0% of events were classified as “unknown,” highlighting the challenge of determining the cause of a device malfunction or adverse event. In addition, 4.6% of events used terms that were not included in the Medical Device Problem Terminology, underscoring the importance of reporting according to standardized terminology. Although reporting with the appropriate terms is generally required, reports using non-standard terms were occasionally submitted shortly after the terminology was introduced or for rare events. With the lack of international guidelines regarding this issue [20], it is important to develop comprehensive terminologies capable of classifying device malfunctions and adverse events [42].

In this study, CPA was applied to the number of endoleak events for aortic stent grafts. However, further investigations are needed to determine whether similar estimation of confidence levels and change points is possible when applied to other medical devices and different types of device malfunctions or adverse events. Studies should also verify whether CPA can be comprehensively applied to device malfunction reports, particularly for events with few events or limited reporting time points.

## Conclusions

This study investigated the number of device malfunctions related to endoleaks in aortic stent grafts. CPA was applied to identify the time points at which confidence levels exceeded the predefined threshold and the change points were detected. The analysis results suggested that these signals emerged approximately one to two years before the issuance of the “PMDA Alert for Proper Use of Medical Devices.” The findings suggest that CPA may serve as a complementary tool for monitoring temporal changes in adverse event reporting and help support the prioritization of investigations into medical-device-related malfunctions and adverse events. However, as CPA does not directly reflect the risk thresholds or broader decision-making processes underlying regulatory alert issuance, these results should not be interpreted as evidence of earlier or superior signal detection compared with regulatory authorities. Furthermore, the absence of device exposure or utilization data is a key limitation. In this context, CPA reflects changes in reporting frequency rather than true changes in risk, which may be influenced by variations in device usage or reporting behavior. Further research is needed as CPA has not yet been applied to other medical devices or other types of device malfunctions or adverse events.

## Data Availability

The datasets generated and analyzed during the current study were provided as part of an AMED-funded research project and are not publicly available. However, data can be requested through information disclosure system of the PMDA; although the level of detail may vary, access can be granted upon such request (Request for Disclosure of Corporate Documents).
